# Effects of virtual reality simulation on medical students’ learning and motivation in human parasitology instruction: a quasi-experimental study

**DOI:** 10.1186/s12909-023-04589-3

**Published:** 2023-09-03

**Authors:** Fei Gao, Jingfan Qiu, Lin Chen, Lan Li, Minjun Ji, Rong Zhang

**Affiliations:** 1 Department of Visual Communication and Technology Education, Bowling Green State University, Bowling Green, OH USA; 2 Department of Pathogen Biology, Key Laboratory of Pathogen Biology of Jiangsu Province, Nanjing Medical University, Nanjing, China; 3 School of Teaching and Learning, Bowling Green State University, Bowling Green, OH USA; 4 National Demonstration Center for Experimental Basic Medical Education, Nanjing Medical University, Nanjing, China

**Keywords:** Virtual reality simulation, Motivation, Medical education

## Abstract

**Background:**

Despite the proven effectiveness of simulation-based learning activities, its adoption in medical education remains limited, and the influence of simulation on student motivation, particularly subjective task values, is seldom explored. This study aimed to investigate the impact of a simulation-based learning activity on student learning and subjective task values in a medical morphology-related course of Human Parasitology.

**Methods:**

A quasi-experimental study was conducted with 113 Chinese undergraduate medical students who participated in a Human Parasitology course during April to May 2022. Students were divided into two groups: Simulation Group (n = 55), where students used the simulation, and Lecture Group (n = 58), where students attended an online lecture. Students’ learning was measured prior to the intervention, immediately after the intervention, and three weeks later to assess knowledge retention. The subjective task values questionnaire was administered before and after the interventions. Data were analyzed using one-way ANCOVA and MANOVA.

**Results:**

Students in the Simulation Group exhibited significantly higher knowledge gain compared to the Lecture Group [*F* (1,110) = 23.69, *p* < 0.01]. Additionally, the Simulation Group retained knowledge significantly better than the Lecture Group [*F* (1,101) = 10.05, *p* < 0.005]. Furthermore, students in the Simulation Group experienced a significant increase in subjective task values after the intervention [*F* (3, 52) = 3.57, *p* < 0.05, *η*_*p*_^*2*^ = 0.17], while students in the Lecture Group reported a significant decrease in subjective task values [*F* (3, 55) = 2.96, *p* < 0.05, *η*_*p*_^*2*^ = 0.14].

**Conclusions:**

Simulation-based learning not only leads to superior learning but also enhances students’ subjective task values. These findings offer valuable insights into designing effective simulation-based learning experiences in medical education and have significant practical implications for educators and medical professionals.

**Supplementary Information:**

The online version contains supplementary material available at 10.1186/s12909-023-04589-3.

## Background

Medicine is a highly practical subject, where experimental teaching plays an important role in cultivating the practical ability and clinical thinking of medical students [1]. However, four challenges exist in the current medical experiment teaching. (1) Increased experimental costs: As the number of students increases, more experimental materials, instruments, and animals are needed, which results in increased costs. (2) Medical ethics and environmental protection requirements: There is a need to reduce the consumption of experimental materials and animals as much as possible to meet the requirements of medical ethics and environmental protection. (3) Biosafety limitations: Some experiments with pathogenicity, infectivity or trauma cannot be carried out due to legislation and regulations related to safety and security of biological materials. (4) Demand for knowledge integration: Medicine is a comprehensive subject emphasizing the integration and application of interdisciplinary knowledge, but experiments involving knowledge integration are difficult to carry out in the classroom [2].

### Virtual reality simulation and medical education

Virtual reality simulation provides an alternative way to address the above-mentioned challenges in medical experiment teaching. Virtual reality simulation refers to the construction of various virtual experimental environments on a computer to simulate real experimental scenarios [3]. Experimenters can use virtual instruments and equipment to perform virtual operations on experimental animals, specimens, or human organs as in the real experiment. They then, complete a variety of scheduled virtual experimental projects and gain an immersive learning experience [4]. Virtual reality simulation was initially applied in military, business, and academic laboratories. With the development of science and technology and the reduction of application cost, virtual simulation has been gradually applied in the field of education. The modern era of medical simulation has its origins in the second half of the 20th century [5]. Medical simulations range widely in fidelity and realism from simple task trainers to manikins, multimedia computer systems and standardized patients [6]. Issenberg et al. identified 10 features of medical simulations as effective educational interventions: (1) providing feedback, (2) repetitive practice, (3) curriculum integration, (4) range of difficulty level, (5) multiple learning strategies, (6) clinical variation, (7) controlled environment, (8) individualized learning, (9) defined outcomes, and (10) simulator validity [7]. Simulations allow medical learners to master medical knowledge and practice clinical skills under safe, controlled, forgiving conditions with the goals of acquiring and maintaining clinical competence [7, 8]. Medical education research spanning at least four decades demonstrates that simulation technology has large and sustained effects on knowledge and skill acquisition and retention among medical learners. In comparison with either no intervention or other instructional methods, simulation-based medical education improves the knowledge, procedural skills, behavior, teamwork and communication of medical students [9, 10]. Although existing research literature has shown positive effects of simulation on learning in clinical medical education [9, 11], few studies have been conducted to examine the effectiveness of simulation on teaching medical morphology-related topics.

Human Parasitology is an important subject in the field of basic medicine, encompassing not only the understanding of pathogen morphology but also practical competencies related to laboratory diagnosis and disease prevention and control. However, due to the inherent risk of infection and the restricted geographical distribution of certain diseases, the traditional approach to practical training in investigating and preventing parasitic diseases is limited. As a solution, in this study, a virtual reality simulation called “Schistosomiasis Prevention and Control” has been developed and integrated into the experimental teaching of Human Parasitology to overcome the limitations of traditional experimental teaching methods. By immersing students in a simulated environment, it provides a safe and controlled platform to acquire practical skills and knowledge essential to combating parasitic diseases.

### Motivation, subjective task values, and learning

Motivation is a major determinant of the quality of learning and is of particular importance in highly professional areas such as health sciences [12]. It involves learners’ beliefs about their competence and expectancies for success and task values, and directly affects their persistence and effort in performing academic tasks [13]. Despite its importance, motivation and other affective dimensions of learning has often been neglected and under-evaluated in simulation-based medical education research [14]. Studies examining simulation’s effects on medical students’ affective dimension of learning are limited [15]. According to expectancy-value theory, individuals’ motivation to perform a task depends on two components: expectancies for success and subjective task values [16, 17]. Expectancies for success are individuals’ beliefs about how well they will do on a task. Subjective task values consist of three components: (1) Importance: the attainment value of the task; (2) Interest: the intrinsic or interest value of the task; and (3) Usefulness: the utility value of the task for future goals [17]. Empirical studies have consistently suggested that subjective task values play an important role in the development of interest [18], and it either predicts or is strongly related to student academic performance [19–21]. As a result, it is important to have learners work on tasks that not only improve their learning but also enhance their perceptions of task values so they are more motivated to learn.

## Methods

### Aims

The current study attempts to address two gaps in virtual reality simulation research in medical education: (1) lack of research on teaching medical morphology-related topics and (2) lack of motivation-related research. We examined the effectiveness of a 3D virtual reality simulation in improving student learning and a critical aspect of students’ motivation, subjective task values, when they studied a medical morphology-related topic: Human Parasitology. A quasi-experimental study was conducted to determine the effects of the simulation intervention on students’ learning and subjective task values. The objective of the study was to test three hypotheses:

1. Students in the simulation-based learning group perform better than those in the traditional lecture-based instruction group in the learning test immediately after the intervention.
2. Students in the simulation-based learning group perform better on a delayed retention test than those in the traditional lecture-based instruction group.
3. Simulation-based learning has more positive impact on student subjective task values than traditional lecture-based instruction.

### Participants

Participants were 113 s-year medical school students from four sections of the same Human Parasitology course in a medical university in China during April to May 2022. All participants were informed of the aims, methods, potential benefits and risks of the study, and signed the consent forms. The study has been reviewed and approved by the Institutional Review Board of the university.

### Virtual reality simulation

**Fig. 1:**
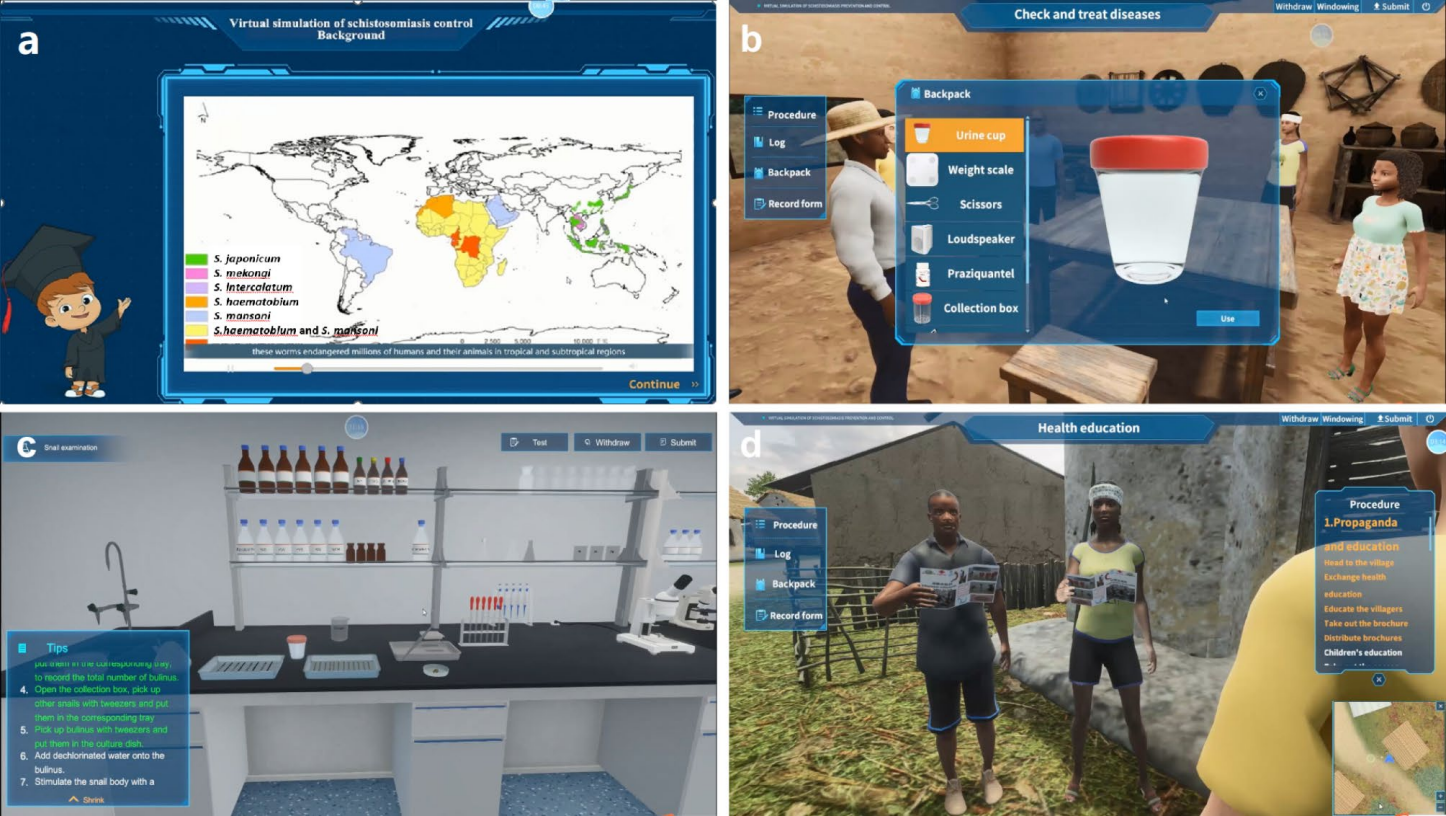
Screenshots of the Simulation. **a** Background, **b** Check and treat diseases, **c** Detection and elimination of snails, **d** Health education

The virtual reality simulation of “Schistosomiasis Prevention and Control” simulated the epidemic scene of schistosomiasis in Africa. As presented in Fig. 1., there were four modules in this simulation: (1) Background, (2) Check and treat diseases, (3) Detection and elimination of snails, and (4) Health education. In each module, students viewed the content on a computer screen and operated step by step according to the instructions such as collecting the villagers’ urine samples, detecting the eggs of parasites, providing medication and so on. At the end of each module, students answered a few multiple-choice questions to check their understandings of the content in the module. It took about one hour to complete the entire virtual reality simulation activity. Upon completion of the simulation, students are provided with a comprehensive performance assessment, including a score evaluation and an analysis of their knowledge gaps and deficiencies.

### Learning tests

Three learning tests were administered in the study. First, a prior knowledge test, which was administered prior to the intervention, consisted of 10 multiple choice questions, with 5 points per question, totaling 50 points. The test was to assess students’ prior knowledge on relevant topics including epidemic distribution, life cycle, pathogenicity, laboratory diagnosis, treatment, and prevention of schistosomiasis. Second, a post-intervention test, which was administered at the end of the intervention to test what students had learned, consisted of 20 multiple choice questions, with 5 points per question, totaling 100 points. Of the 20 questions, 15 questions assessed students’ declarative knowledge of schistosomiasis, and 5 questions assessed students’ procedural knowledge related to diagnosis and control of schistosomiasis. Third, a delayed retention test that was identical to the post-intervention test was used three weeks after the intervention to measure student delayed retention of learning.

### Subjective task values questionnaire

The subjective task values questionnaire was used to measure students’ perceived value of learning Human Parasitology. It was developed based on Cole et al. [20], Glynn et al. [22], Velayutham et al. [23], and Bryan et al.’s [24] work on measuring student learning task value in science learning. The questionnaire consists of three subscales: (1) Interest, (2) Usefulness, and (3) Importance. Under each of the subscales, there are three seven-scale Likert questions asking students to rate the value of learning Human Parasitology. Some example questions are “I am very interested in learning Human Parasitology”, “I think that the materials are useful for me to learn”, and “It is important for me to learn the materials in this class” (See Supplementary file 1). The internal consistency reliability for each scale was later assessed using Cronabach’s alpha, and the values for interest, importance, and usefulness scales were 0.954, 0.918, and 0.935 respectively. The subjective task values questionnaire was administered twice, one prior to the intervention, and the other at the end of the intervention. Three additional open-ended questions were added to the one administered at the end of the intervention, asking students to explain whether and how they thought the design of the learning activity positively or negatively affected their motivation and their learning, and how they thought the learning activity could be improved.

### Procedures

**Fig. 2:**
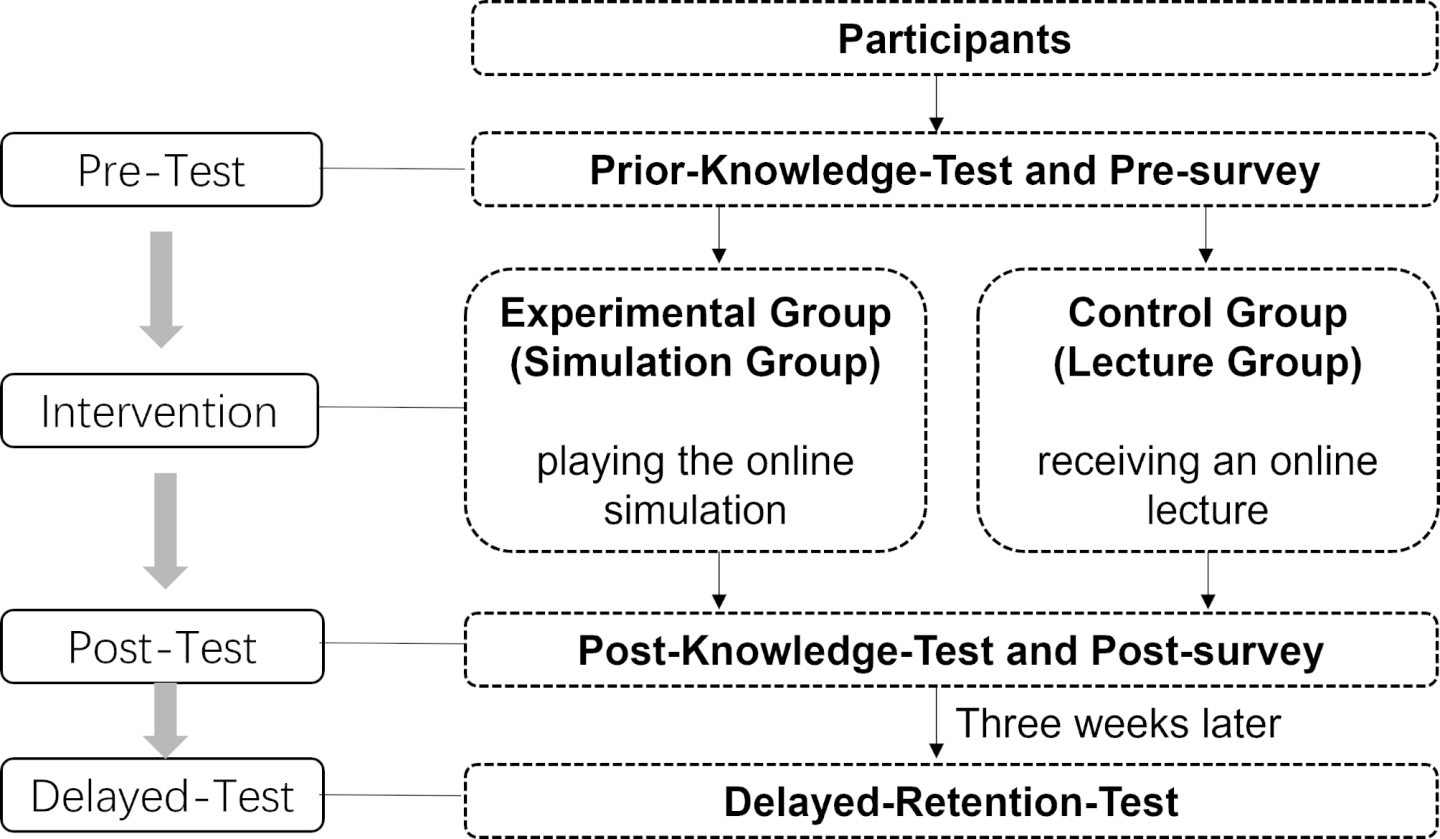
The procedures of the study

The procedures of this study were shown in Fig. 2. Prior to the intervention, all students took the prior knowledge test and the pre-survey of subjective task values. During the intervention, students in all sections were taught by the same instructor on the topic of the prevention and control of a parasitic disease, schistosomiasis. The intervention involved the learning of basic knowledge, clinical diagnosis and treatment skills related to schistosomiasis. Students in Sects. 1 & 2 were assigned to the experimental group (Simulation Group), where they used the simulation. Students in Sects. 3 & 4 were assigned to the control group (Lecture Group), where they received an online lecture. The same content was taught in both groups but in different ways. In the Simulation Group, the instructor first presented the background knowledge of preventing and controlling schistosomiasis online using PowerPoint Slides. Then, students worked individually on the simulation completing a mission where they helped people in a small African village prevent and control schistosomiasis. Students completed the simulation activity only once, and there was no opportunity to revise their responses or repeat the activity. In the online Lecture Group, the instructor presented the background knowledge of preventing and controlling schistosomiasis as well as the procedures of how to prevent and control schistosomiasis online using PowerPoint Slides. Then, students watched a video clip showing a real-life case of preventing and controlling schistosomiasis in Zanzibar. After that, students responded to five multiple-choice questions that prompted them to reflect on the content learned. At the end of the intervention, all participating students completed the post-intervention test and the post-survey of subjective task values. Three weeks after the intervention, all students took a delayed retention test that was identical to the post-intervention test. A delay of three weeks was selected to represent a typical period of length in delayed retention testing ( > = 2 weeks) in the literature [25, 26]. Students were not told about the delayed retention test during the intervention. After the delayed retention test, students in the Lecture Group completed the virtual reality simulation in order to receive the benefits of using the simulation.

### Statistical analysis

To compare student learning and delayed retention between the experimental and control group, a one-way ANCOVA was conducted. Condition was set as the independent variable, students’ post-intervention test score or delayed retention test score as the dependent variable, and students’ prior knowledge test scores were used as covariance. In the subjective task values questionnaires, student responses to Likert-scale questions were coded into numbers ranging from 1 (strongly disagree) to 7 (strongly agree). Student scores on each of the three subscales were calculated by adding their ratings of all three items in the subscale. The differences between the pre-intervention and post-intervention scores in each subscale were also calculated. Since correlations exist among the three subscales, multivariate analysis of variance (MANOVA) was used to increase the statistical power and control for Type I error. Follow-up ANOVAs were then conducted to identify the significance level of the differences. All analyses were conducted using IBM SPSS Statistics 25.0, and statistical significance was defined as a p-value less than 0.05.

## Results

### Basic characteristics of participants

Among the 113 participants, 67 (59.3%) are female and 46 (40.7%) are male. They were between 18 and 22 years old with a mean age of 19.87 and a standard deviation of 1.11. Basic characteristics of the students participating in the two groups are presented in Table 1.

**Table 1:** Basic characteristics of participants

	Simulation Group (Experimental Group)	Lecture Group (Control Group)
Number	55	58
Gender		
Female	34 (61.81%)	33 (56.90%)
Male	21 (38.18%)	25 (43.10%)
Age, mean (SD)	19.89 (1.42)	19.84 (0.72)

### Knowledge acquisition

As presented in Table 2, there was a significant difference in students’ post-intervention test scores between the experimental and control group when prior knowledge test scores were controlled [*F* (1,110) = 23.69, *p* < 0.01], suggesting students in the Simulation Group gained significantly more knowledge than those in the Lecture Group.

**Table 2:** ANCOVA Results and Descriptive Statistics for Post-Intervention Test Scores (n = 113)

Condition	Mean (SD) of Students’ Prior-Knowledge-Test Score			Mean (SD) of Students’ Post-Intervention-Test Score			N
Simulation Group	32.00 (9.00)			85.18 (9.52)			55
Lecture Group	37.32 (5.56)			74.48 (14.13)			58
**Source**		**SS**	**DF**		**MS**	**F**	
Prior Knowledge Test Score		244.30	1		244.30	1.68	
Condition (Group)		3454.52	1		3454.52	23.69 **	
Error		16038.36	110		145.80		

SD standard deviation; ** *p* < 0.01

### Delayed retention

Among the 113 students, 104 took the delayed retention test. The results are presented in Table 3, where a significant difference between the two groups was found [*F* (1,101) = 10.05, *p* < 0.005], indicating students in the Simulation Group retained their knowledge significantly better than those in the Lecture Group.

**Table 3:** ANCOVA Results and Descriptive Statistics for Delayed Retention Test Scores (n = 104)

Condition	Mean (SD) of Students’ Prior-Knowledge-Test Score			Mean (SD) of Students’ Delayed-Retention-Test Score			N
Simulation Group	32.00 (9.00)			69.51 (12.82)			51
Lecture Group	37.32 (5.56)			61.42 (16.71)			53
**Source**		**SS**	**DF**		**MS**	**F**	
Prior Knowledge Test Score		621.70	1		621.70	2.84	
Condition (Group)		2200.98	1		2200.98	10.05 ***	
Error		22109.92	101		218.91		

SD standard deviation; *** *p* < 0.005

### Subjective task values

The results of the MANOVA, as presented in Table 4, showed that, for the Simulation Group, there was a significant increase on student subjective task values after the intervention [*F* (3, 52) = 3.57, *p* < 0.05, *η*_*p*_^*2*^ = 0.17]. The follow-up ANOVAs showed that the difference in the Importance subcategory reached the significance level of *p* = 0.0498. Though the difference in the Interest and Usefulness subscales did not reach the significance level, Simulation Group students gave higher ratings of Interest in the post-survey than in the pre-survey. This is probably due to a ceiling effect – as indicated in Table 3, Simulation Group students had a relatively high subjective task values for each subscale (close to the full score of 21) prior to the intervention. For the Lecture Group, there was a significant decrease in student subjective task values after the lecture [*F* (3, 55) = 2.96, *p* < 0.05, *η*_*p*_^*2*^ = 0.14]. The follow-up ANOVAs showed that the differences of scores in each subscale were significantly lower on the post-scores than the pre-scores.

**Table 4:** MANOVA Results on the Differences in Subjective Task Values Scores (n = 113)

Condition	Subscale	Pre-Scores (M&SD)	Post-Scores (M&SD)	Difference (M&SD)	F	*p*	*η*_*p*_^*2*^
Simulation Group (n = 55)					3.57	0.020	0.17
	Interest	17.62 (2.98)	18.40 (2.66)	0.78 (3.41)	2.89	0.095	0.05
	Importance	18.33 (2.69)	19.09 (1.99)	0.76 (2.82)	4.03	0.0498	0.07
	Usefulness	19.04 (2.08)	18.98 (2.12)	− 0.05 (2.50)	0.03	0.872	0.00
Lecture Group (n = 58)					2.96	0.040	0.14
	Interest	18.28 (2.84)	17.34 (3.43)	− 0.93 (3.16)	5.03	0.029	0.08
	Importance	19.03 (2.24)	18.07 (2.57)	− 0.97 (2.59)	8.07	0.006	0.12
	Usefulness	19.22 (2.14)	18.26 (2.40)	− 0.97 (2.53)	8.42	0.005	0.13

M mean; SD standard deviation

## Discussion

The objective of the present study was to investigate whether simulation-based learning leads to superior learning and enhanced subjective task values when used to teach medical students the topic of Human Parasitology. A quasi-experimental study was conducted, where students were assigned to either of two conditions, a Simulation Group or a Lecture Group. Student learning during the intervention was measured both immediately after the intervention and three weeks later. Student subjective task values were measured both prior to and after the interventions.

The results of the quasi-experiment showed that students in the Simulation Group gained significantly more knowledge than those in the Lecture Group. Moreover, students in the Simulation Group retained their knowledge significantly better than those in the Lecture Group. These findings are consistent with those from previous studies on simulation-based learning [9, 10]. A possible reason for the superior learning outcomes in simulation-based learning is that simulation-based learning allows students to interact with the virtual world and have active hands-on practice. According to Kolb’s Experiential Learning Theory [27], there are four stages of the learning cycle: concrete experience, reflective observation, abstract conceptualization, and active experimentation. In our 3D virtual reality simulation, students were able to engage in a simulation scenario to gain concrete experience and perform a series of procedures to accomplish the mission through active experimentation. Such learning opportunity was missing in traditional lecture-based instruction. This is consistent with students’ responses to the open-ended questions, where students commented that the simulation-based learning activity is “realistic”, “authentic”, and “interactive”.

The results also suggested that simulation-based learning had a positive impact on student subjective task values, while the lecture-based instruction had a negative impact on student subjective task values. The positive effect of simulation-based learning can be explained by the motivation-enhancing feature of simulation. According to Hofstede GJ, Caluwe LD, Peters V [28], simulation games are a powerful tool to raise people’s awareness of the importance of an issue, and thus motivates them to seek knowledge and skills that they lack. In our 3D virtual world simulation, students were able to apply what they had learned to accomplish a mission by successfully helping the villagers, so students could easily see the value of their learning. This is also reflected in students’ survey responses, as a student wrote that “accomplishing the mission in the simulation helped me understand that what I have learned from class can be directly applied to our lives.” We were surprised, however, to find that students in the Lecture Group rated the task values significantly lower after they received the online lecture. When designing and delivering the online presentation, we made every effort to present the information in a fun and easy-to-understand manner. We also intentionally included a video to show students the relevance of their learning and promote their interest. To gain insight into this result, we reviewed student comments to the open-ended questions in relation to their ratings on subjective task values. While many students commented positively that the real case presented in the video increased or supported their interest and the instructor presented the content in a fun and relevant way, many commented that there was a lack of hands-on activities in the teaching process. This suggests that the Lecture Group’s lower post-survey ratings on subjective task values may be attributed to limited active participation and interaction during the learning process.

The findings make a significant contribution to the existing literature in two ways. First, the results further confirmed the effectiveness of simulation in teaching declarative and procedural knowledge in medical education [15, 29] and suggested that simulation-based learning can be effectively adopted to teach a wide range of content including Human Parasitology. Second, the study is one of the first few studies that examined the effects of simulation-based learning on medical students’ subjective task values, which is a critical component of motivation to learn. The different impacts on students’ subjective task values between the Simulation Group and Lecture Group suggested that simulation, when designed properly, has a potential to affect not only students’ learning but also their motivation. This finding is important because students’ motivation plays a central role in learning and performance and is considered an enabler for academic success [30]. As a result, this study calls for future research on using simulation in medical education to increase students’ motivation to learn. We are currently conducting follow-up studies to understand how specific aspects of simulation impact medical students’ intrinsic motivation by satisfying their basic psychological needs for autonomy, competence and relatedness [31], the findings of which will provide additional guidance for the design of simulation-based learning in medical education. The findings of the current study also have important practical implications. Medical education has gradually shifted from traditional lectures using a teaching paradigm toward more experience-based approaches using a learning paradigm [32]. Simulation provides a perfect means for such a shift by supporting both students’ learning and motivation.

The study has a few limitations. First, this study employed a quasi-experimental design. Admittedly, quasi-experiments have lower internal validity than true experiments, and future studies with true experimental design are encouraged to validate the findings in this study. However, quasi-experiments can be useful when it is impractical to run a true experiment, which is the case in this study. In addition, quasi-experiments often have higher external validity because the interventions are carried out in real-world settings instead of laboratories. Second, although the study examined students’ immediate learning and delayed retention, it did not examine the process of learning. A valuable direction for future research is to study more closely how students learn in both conditions, so that we could gain insights on what causes the differences in student learning and motivation. Third, it is important to acknowledge the potential novelty effect, that is, the tendency for performance to initially improve when new technology is adopted. It is, therefore, important to determine the long-term effects of such 3D virtual reality simulations on learning through longitudinal research. Fourth, this study examined the impact of virtual reality simulation on student learning and subjective task values in a course on Human Parasitology. Future research is still needed to further study the impact of virtual reality simulation on student learning of other medical morphology-related courses, such as Histoembryology, Pathology, Microbiology, and so on. Such research will provide additional insights on how to apply virtual reality simulation to improve the quality of basic medical education.

## Conclusions

The incorporation of virtual reality simulation in the teaching of Human Parasitology opens up new possibilities for effective and engaging learning experiences. It enables students to actively participate in such activities as laboratory diagnosis and disease prevention, which were previously challenging to replicate in traditional classroom settings. By bridging the gap between theory and practice, this approach has the potential to revolutionize the experimental teaching of Human Parasitology.

Our study demonstrates that simulation-based learning is more effective in improving students’ learning as compared to the traditional lecture-based instruction when used to teach medical students the topic of Human Parasitology. In addition, simulation-based learning enhances student subjective task values, while the traditional lecture-based instruction has a negative impact on student subjective task values. Our findings provide important insights on the design of simulation-based learning and call for additional research on the adoption of virtual reality simulation in medical education.

### Electronic supplementary material

Below is the link to the electronic supplementary material.

**Supplementary file 1**: Subjective Task Values Questionnaire

## Data Availability

The datasets during and/or analyzed during the current study are available from the corresponding author on reasonable request.

## Abbreviations

M: Mean
SD: Standard Deviation
