# Supplementary material for: Effects of virtual reality simulation on medical students’ learning and motivation in human parasitology instruction: a quasi-experimental study
Source: BMC Med Educ. 2023 Sep 3;23:630. doi: 10.1186/s12909-023-04589-3 (PMC10476417; doi:10.1186/s12909-023-04589-3)
Supplement: Supplementary file 1 — Subjective Task Values Questionnaire [file 12909_2023_4589_MOESM1_ESM.docx]

**Subjective Task Values Questionnaire**

Based on your experience with the simulation, please rate the statements below using the following scales:

- Strongly Disagree
- Disagree
- Somewhat Disagree
- Neutral
- Somewhat Agree
- Agree
- Strongly Agree

**Interest**

- I am very interested in learning Human Parasitology.
- I like learning Human Parasitology.
- Learning parasites is fun to do.

**Usefulness**

- I think that I will be able to use what I learn.
- I think that the materials are useful for me to learn.
- I think what I learn is of practical value.

**Importance**

- It is important for me to learn the materials in this class.
- Understanding the subject matter of this course is very important to me.
- It is important to me to improve my skills in this course.
